# Clinical utility of postprocessed low-dose radiographs in skeletal imaging

**DOI:** 10.1259/bjr.20210881

**Published:** 2022-01-05

**Authors:** Johannes Kolck, Katharina Ziegeler, Thula Walter-Rittel, Kay Geert A. Hermann, Bernd Hamm, Alexander Beck

**Affiliations:** 1 Department of Radiology, Charité - Universitätsmedizin Berlin, Berlin, Germany

## Abstract

**Objectives:**

Radiography remains the mainstay of diagnostic and follow-up imaging. In view of the risks and the increasing use of ionizing radiation, dose reduction is a key issue for research and development. The introduction of digital radiography and the associated access to image postprocessing have opened up new opportunities to minimize the radiation dosage. These advances are contingent upon quality controls to ensure adequate image detail and maintenance of diagnostic confidence. The purpose of this study was to investigate the clinical applicability of postprocessed low-dose images in skeletal radiography.

**Methods:**

In our study setting, the median radiation dose for full dose X-rays was 9.61 dGy*cm2 for pelvis, 1.20 dGy*cm2 for shoulder and 18.64 dGy*cm2 for lumbar spine exams. Based on these values, we obtained 200 radiographs for each anatomic region in four consecutive steps, gradually reducing the dose to 84%, 71%, 60% and 50% of the baseline using an automatic exposure control (AEC). 549 patients were enrolled for a total of 600 images. All X-rays were postprocessed with a spatial noise reduction algorithm. Two radiologists assessed the diagnostic value of the radiographs by rating the visualization of anatomical landmarks and image elements on a five-point Likert scale. A mean-sum score was calculated by averaging the two reader’s total scores. Given the non-parametric distribution, we used the Mann-Whitney U test to evaluate the scores.

**Results:**

Median dosage at full dose accounted for 38.4%, 48 and 53.2% of the German reference dose area product for shoulder, pelvis and lumbar spine, respectively. The applied radiation was incrementally reduced to 21.5%, 18.4% and 18.7% of the respective reference value for shoulder, pelvis and lumbar spine. Throughout the study, we observed an estimable tendency of superior quality at higher dosage in overall image quality. Statistically significant differences in image quality were restricted to the 50% dose groups in shoulder and lumbar spine images. Regardless of the applied dosage, 598 out of 600 images were of sufficient diagnostic value.

**Conclusion:**

In digital radiography image postprocessing allows for extensive reduction of radiation dosage. Despite a trend of superior image detail at higher dose levels, overall quality and, more importantly, diagnostic utility of low-dose images was not significantly affected. Therefore, our results not only confirm the clinical utility of postprocessed low-dose radiographs, but also suggest a widespread deployment of this advanced technology to ensure further dose limitations in clinical practice.

**Advances in knowledge:**

The diagnostic image quality of postprocessed skeletal radiographs is not significantly impaired even after extensive dose reduction by up to 20% of the reference value.

## Introduction

Despite the increasing availability of cross-sectional techniques, plain radiographs remain the most commonly used tool in diagnostic and follow-up imaging. In 2014 alone, about 140 million X-rays were taken in Germany, of which skeletal images accounted for the second-largest share.^1^ Recent data from England show that conventional imaging, with 23.2 million X-rays, is still used considerably more frequently than cross-sectional imaging.^2^ Main benefits of X-ray diagnostics are the high availability, low cost, fast acquisition time and the portability of X-ray equipment.^3^ The introduction of digital radiography represents a milestone in conventional imaging, which brought further advances in image quality and dose reduction. Due to the frequent usage and known risks of ionizing radiation, dose minimization continues to be a focus in research and development.^4–8^ In this respect, there are several levers to implement the as low as reasonably achievable (ALARA) principle:

One is hardware optimization, which has already contributed to reduced radiation in various settings. Guo et al optimized image detail and applied dose by adjusting the tube voltage in pediatric chest imaging depending on patient age, thus reducing dosage by 18.75% compared to the control group.^9^ Kloth et al implemented varying exposure classes for quality-controlled dose reduction in full-leg imaging and in follow-up after hip and knee arthroplasty. Focused diagnostic confidence was maintained, even with an increase in image noise.^10–12^ Ernst et al used an automatic exposure control device for dose reduction of full-spine radiographs in pediatric patients with idiopathic scoliosis.^13^ Also, the application of advanced technologies, such as biplanar low-dose X-ray systems, allowed dose reduction in full-length lower limb and whole spine radiography.^14^

A newer pillar in digital radiography is image postprocessing. Unlike conventional film radiography, digital images can be edited after acquisition. This allows the implementation of complex algorithms capable of enhancing low-dose radiographs to standard-dose quality.^15,16^ Corresponding results have been reported from Ziegeler et al for rheumatic hand imaging and from Lee et al for chest X-rays.^17,18^

The purpose of the present prospective study is to evaluate the clinical applicability of postprocessed low-dose images in orthopedic and trauma radiography.

## Methods and materials

### Experimental setup

Our university hospital is one of five certified trauma centers in the Berlin-Brandenburg region. In trauma, orthopedic and preoperative imaging, pelvis, shoulder and lumbar spine are among the most frequently X-rayed parts of the skeleton. These anatomic regions are particularly interesting for two other reasons: Firstly, radiographs of the pelvis and lumbar spine require protection of the reproductive organs, which are specifically sensitive to radiation. Secondly, conventional imaging of the pelvis, lumbar spine and shoulder is often more challenging due to the usually thicker soft tissue layer in these regions. Therefore, we decided to acquire radiographs of the pelvis, shoulder, and lumbar spine with 84%, 71%, 60%, and 50% reduced radiation dose, respectively.

According to the specifications of the Federal Office for Radiation Protection in Germany, the rDAP is composed of nationwide exams of outpatient and hospitalized patients. The guideline states, that the mean dosage over at least ten arbitrarily selected patients with different body dimensions should not exceed the corresponding reference value.^19^ In order to generate data sets that can be benchmarked against the rDAP, we decided to consecutively study 50 patients per dose group and anatomical region without pre-selection.

### Patient collective

Between fall 2017 and 2020, we enrolled 549 patients – 272 female and 277 male – referred to our university hospital for radiographs of either pelvis, shoulder or lumbar spine. The pelvis group comprised 90 female and 93 male patients with an average age of 58.65 years (±18.84). For the shoulder group, 99 females and 84 males with an average age of 63.26 (±17.09) years were examined. The lumbar spine group consisted of 83 female and 100 male patients and had an average age of 59.46 (±18.26) years. Informed consent was obtained from all patients prior to image acquisition. To ensure broad applicability, the patients were examined consecutively without fixed selection criteria. Only legally incompetent or pregnant patients were excluded. This prospective study was approved by the institutional ethics committee in 2017 (submission number EA 2/011/17).

### Imaging technique

Anteroposterior radiographs of the pelvis and shoulder, as well as lateral X-rays of the lumbar spine were acquired on GC85A X-ray unit (Samsung, Seoul, South Korea). In order to control the preservation of diagnostic quality in each dose group and to prevent the acquisition of diagnostically insufficient images, we started with the acquisition of radiographs at 84% of baseline dosage. Once sufficient image quality was confirmed, we reduced the dose to 71% of baseline. In this way, one patient after the other was examined until all 50 X-ray images of the 50% groups had been taken. An Automatic Exposure Control (AEC) was applied to capture low dose images, which works as follows: During image acquisition, radiation exposure is measured on the detector surface. When a selected threshold value is reached, the X-ray generator is switched off via the AEC. This helps to acquire radiographs with a consistent optical density, regardless of the patient’s body shape.^20^Also it enables the acquisition of low-dose images with discrete AEC steps and corresponding thresholds at 84%, 71%, 60% and 50% of the standard dose. For pelvis and lumbar spine X-rays tube voltage was fixed at 77kVp, tube charge was 20 mAs and 16 mAs, respectively. Shoulder images were obtained at 70kVP tube voltage and 8 mAs tube charge. Radiation dosage was recorded as Dose Area Product (DAP in dGy*cm2).

Low-dose image quality was enhanced using a processing algorithm (S-VueTM; Samsung, Seoul, South Korea) in a two-step process. First, a spatially-adaptive noise reduction algorithm obtains local edge information, reduces region specific noise – mainly blurring and scatter noise – and thus minimizes the loss of structural detail. In a second step, the inevitable byproduct of coarse noise is then converted into fine grain noise through a noise whitening process, which further enhances visual performance. The same algorithm was previously implemented and tested in two studies dealing with chest radiography and rheumatoid hand imaging.^18,21^

### Image evaluation and statistical analysis

**Figure 1.**
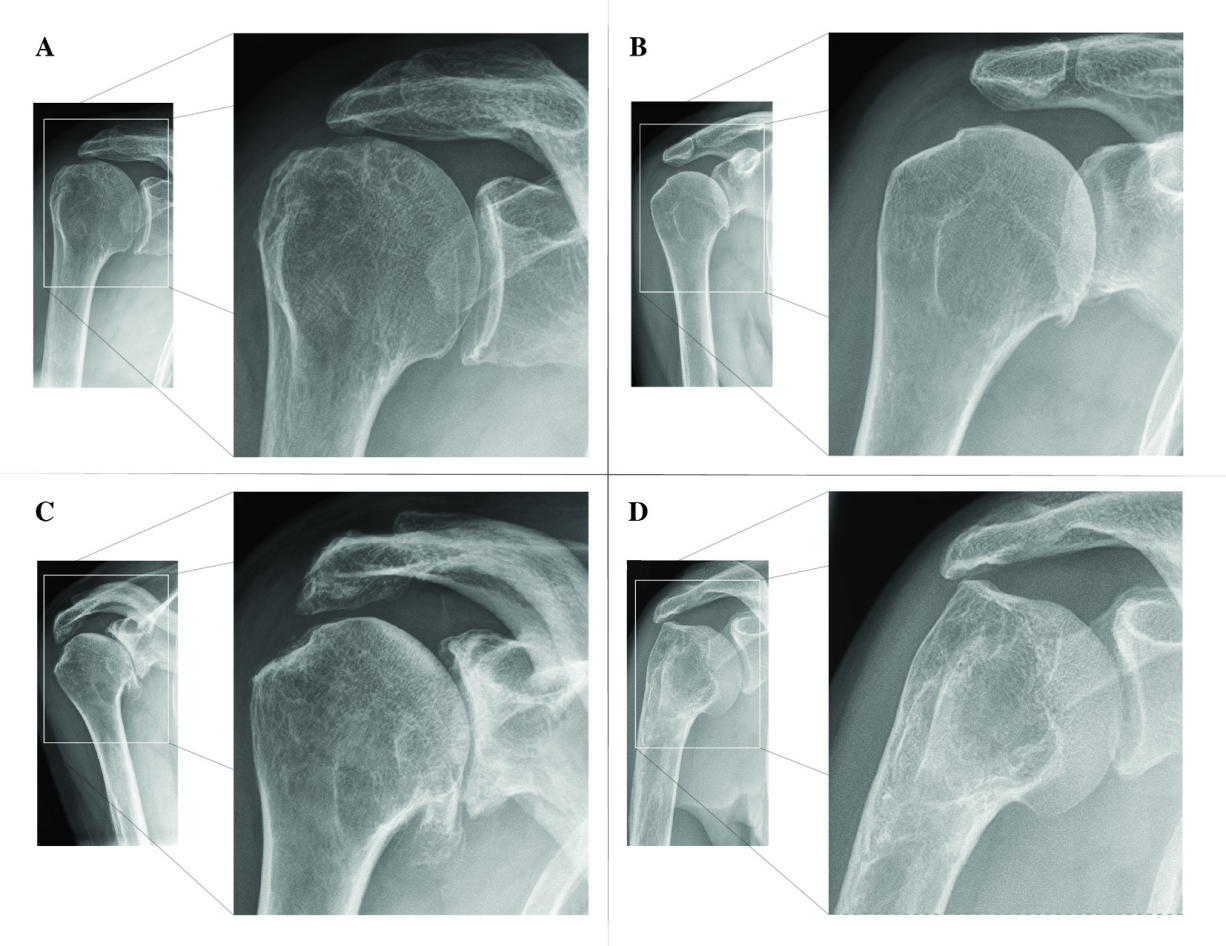
Examples of shoulder radiographs where dose was reduced to 84% (A), 71% (B), 60% (C) and 50% (D) of baseline. Bony cortex, trabeculae, joint spaces, overall contrast and soft tissue visualization were assessed on a five-point Likert scale

All radiographs were randomized and rated by two radiologists with more than three and more than 15 years of experience in musculoskeletal imaging. We adapted a score from Fatouros et al,^22^ to evaluate the radiographs' diagnostic utility in answering clinical questions and visualizing various pathologies. The score incorporates bony cortex, trabeculae, joint spaces, overall contrast and soft tissue. All items were scored semi-quantitively from 1 to 5 (Table 1; Figure 1). The images were read and assessed on dedicated PACS workstations (Centricity PACS, GE Healthcare, Barrington, IL). Windowing and zooming were permitted without restrictions. All images were evaluated in comparison to one full-dose radiographs of the corresponding anatomical region, which showed optimal visualization of the above-mentioned parameters.

**Table 1.** Diagnostic image quality was evaluated according to the items and corresponding grades listed above. This score was adapted from Fatouros et al.^22^

Assessment of image quality by item					
Item	Score				
	1	2	3	4	5
Cortex	Not diagnostic	Poor	Mediocre	Adequate	Optimal
Trabecula	Not diagnostic	Poor	Mediocre	Adequate	Optimal
Joint spaces	Not diagnostic	Poor	Mediocre	Adequate	Optimal
Contrast	Unacceptable	Insufficient	Acceptable	Good	Optimal
Soft tissue	Not diagnostic	Interfering grain	Tolerable grain	Minimal grain	No grain

Overall image performance / diagnostic image quality was assessed with a sum-score of all five items (range 5–25 points). To combine both readers evaluations, we calculated a mean-sum score by averaging the two reader’s total scores per image. Radiographs with a mean-sum score below 15 were considered to be of “minor” quality”. Due to the non-parametric distribution, we used the Mann-Whitney U test to evaluate the mean-sum values. Results were expressed as median scores with interquartile range (IQR) and presented as box plots. For the dose analysis, we randomly selected 50 images of each anatomic region from our database to determine the baseline dose. The Mann-Whitney U test was used for statistical analysis of the dose reduction due to non-parametric distribution. All statistical analyses were performed with Prism 8 (GraphPad Software, San Diego, California) and SPSS, V. 24 (IBM Corporation, New York, USA). Significance was assumed for values with *p* < 0.005. Graphic illustrations were created with Prism eight and Adobe Illustrator (Adobe, San Jose, California).

## Results

### Dose reduction

**Figure 2.**
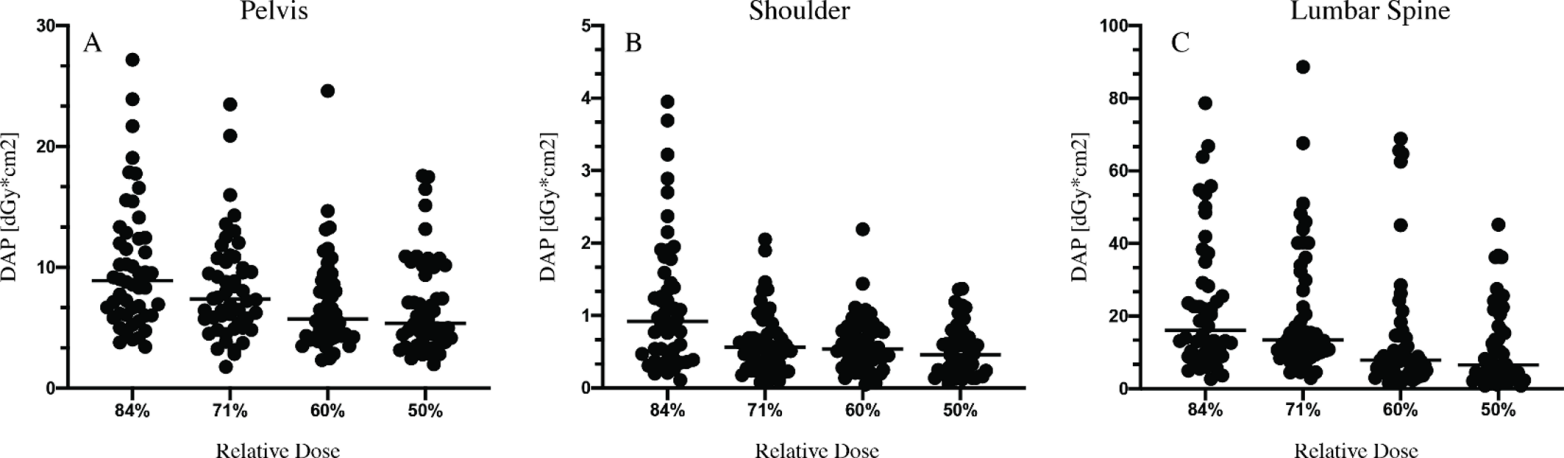
Applied radiation dose per patient measured as dose area product (DAP). Examined were 50 persons per dose group. Median values are represented as bars

The German reference dose area product (rDAP) for ap/pa radiographs of the pelvis and shoulder and for lateral radiographs of the lumbar spine is 25 dGy*cm2, 2.5 dGy*cm2 and 35 dGy*cm^2^, respectively.^19,23^ In our study the median dose at full dose was 9.61 dGy*cm2 (equivalent to 38.4% of rDAP) for pelvis, 1.20 dGy*cm2 (equivalent to 48% of rDAP) for shoulder and 18.64 dGy*cm2 (equivalent to 53.3% of rDAP) for lumbar spine X-rays. Based on these DAPs, radiographs were taken with a gradual reduction in dose to 84%, 71%, 60 and 50% of baseline. A graphical overview of these results is shown in Figure 2.

### Pelvis

Median dosage of the 84% group in pelvis radiographs was reduced to 8.91 dGy*cm2 ( *vs* 9.61 dGy*cm2 at standard dose = 92.7%). The 71% dose group had a median DAP of 7.37 dGy*cm2 ( *vs* 9.61 dGy*cm2 at standard dose = 76.7%), it was only 5.73 dGy*cm2 in the 60% group ( *vs* 9.61 dGy*cm2 at standard dose = 59.6%). Reducing the dose by 50% yielded a median dose of 5.37 dGy*cm2 ( *vs* 9.61 dGy*cm2 at standard dose = 55.9%), which is equivalent to 21.5% of the German reference value for pelvis X-rays. Dosage was significantly reduced in the 50% (*p* < 0.00001), the 60% (*p* < 0.00001) and the 71% group (*p* = 0.004).

### Shoulder

The median DAP of standard dose shoulder X-rays was 1.2 dGy*cm2. Median DAP of the 84% dose group was 0.92 dGy*cm2 ( *vs* 1.2 dGy*cm2 at standard dose = 76.7%), at 71% dose it was 0.57 dGy*cm2 ( *vs* 1.2 dGy*cm2 at standard dose = 47.1%). Images acquired at 60% dose had a median DAP of 0.54 dGy*cm2 ( *vs* 1.2 dGy*cm2 at standard dose = 45%), the median value for 50% dose was 0.46 dGy*cm2 ( *vs* 1.2 dGy*cm2 at standard dose = 38.3%). Median DAP at half dose amounts to 18.4% of rDAP for shoulder radiographs in Germany. The applied radiation dosage was significantly reduced in all study groups: In the 50%, the 60% and the 71% group the *p*-value was <0.00001. In the 84% group, the dose reduction was also significant, with *p* = 0.0455.

### Lumbar spine

In the lumbar spine group, the full dose radiographs had a median DAP of 18.64 dGy*cm2. In the 84% group, the median DAP could be reduced to 16.03 dGy*cm2 ( *vs* 18.64 dGy*cm2 at standard dose = 86%). In the 71% group, DAP was minimized to 13.41 dGy*cm2 ( *vs* 18.64 dGy*cm2 at standard dose = 72%). Radiation was further decreased to 7.89 dGy*cm2 ( *vs* 18.64 Gy*cm2 at standard dose = 42.3%) at 60% dose, and to 6.54 dGy*cm2 at half dose ( *vs* 18.64 dGy*cm2 at standard dose = 35.1%). Median dosage at the lowest dose group accounted for 18.7% of rDAP. Radiation was significantly decreased in the 50% (*p* < 0.00001), the 60% (*p* < 0.00001) and the 71% group (*p* = 0.041).

### Overall image quality

**Figure 3.**
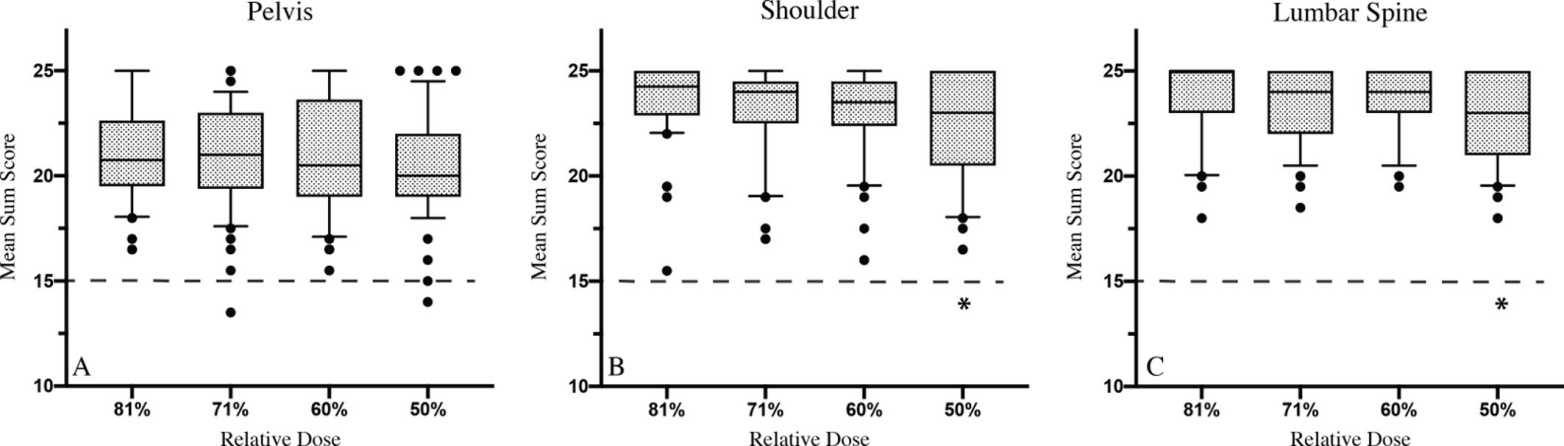
Overall image quality in Mean Sum Scores is visualized as box plots. Median values are represented as bars, significant differences of Mean Sum Scores in comparison to 84% dosage are labelled with *, dashed lines mark the cutoff to “minor image quality”

Across the board, the mean-sum scores correlated with dose reduction – higher image performance was achieved at the cost of higher radiation levels. Significant differences in overall image quality were observed between the 50 and 84% dose group for shoulder (*p* = 0.0055) and lumbar spine (*p* = 0.0192) radiographs. An overview is given in Figure 3.

Median score values for pelvis radiographs ranged between 20 and 21 points. Images at 84%, 71 and 60% dose were rated with 20.75 (IQR 19.5–22.63), 21 (IQR 19.38–23) and 20.5 (IQR 19–23.63) points, respectively. The 50% group had a median score value of 20 (IQR 19–22) points. Only two images were of “minor quality” with 13.5 and 14 points at 71 and 50% dose, respectively.

All shoulder and lumbar radiographs were rated with median values between 23 and 25 points.

At 84% dose, shoulder images reached a median score of 24.25 (IQR 15.5–25), 24 (IQR 17–24.5) points at 71% dose and 23.5 (IQR 16–24.5) points at 60% dose. The 50% group was rated with a median of 23 (IQR 16.5–25) points. Not a single radiograph was of “minor quality”.

Lumbar spine images were assessed with a median value of 25 (IQR 23–25) point at 84% dose. The 71 and 60% dose groups were equally rated with a median of 24 (IQR 22–25; IQR 23–25) points. Radiographs at 50% scored 23 (IQR 21–25) points. Out of all 200 images, none was of “minor quality”.

### Image quality per item

The average score of each image item was calculated for all anatomic regions and dose groups. Additionally, the performance at 71%, 60 and 50% dose was compared to the item visualization at 84% dose (Table 2). There were no significant differences throughout the dose groups in pelvis radiographs. The comparison of shoulder images at 50% *vs* 84% dose showed significantly superior median score values for trabecula (*p* = 0.0051), joint space (*p* = 0.0479), overall contrast (*p* = 0.0156) and soft tissue (*p* = 0.0079) at the 84% dose level. The visualization of soft tissue was also rated significantly higher at the 84% *vs* 60% dose level (*p* = 0.0434). Significant deviations in lumbar spine images were noted in 84% dose images, which surpassed the visualization of joint spaces (*p* = 0.0278) and overall contrast (*p* = 0.0098) at the minimum dose setting (50%). All significantly inferior items still exceeded a score of 4 out of 5 points. Overall, soft tissue in pelvic images was the only item, which was rated with less than four points (3.93 points at 84% dose and 3.81 points at 50% dose). Across all anatomic regions, we observed a decent visualization of foreign materials, ranging from a minimum of 4.35 to a maximum of 4.55 points, and no statistically significant differences between dose groups.

**Table 2.** Shown are the average scores per image item across all 50 radiographs, as rated on a 5-point-Lickert scale by two readers. The performance of each dose group was compared to the quality at 84% dose, reported as p (vs. 84%). Statistically significant differences are printed in bold. SD indicates the standard deviation of the average values per item

Image item visualization													
		Shoulder				Pelvis				Lumbar Spine			
Items	Dose	84%	71%	60%	50%	84%	71%	60%	50%	84%	71%	60%	50%
Cortex	Average	4.33	4.29	4.3	4.17	4.77	4.69	4.69	4.59	4.78	4.78	4.81	4.71
	SD	0.53	0.54	0.56	0.55	0.47	0.49	0.55	0.55	0.43	0.41	0.4	0.46
	P ( *vs* 84%)		*p* = 0.7093	*p* = 0.7838	*p* = 0.1418		*p* = 0.4068	*p* = 0.4362	*p* = 0.0816		*p* = 1.000	*p* = 0.7187	*p* = 0.4337
Trabecula	Average	4.31	4.36	4.3	4.14	4.84	4.72	4.7	4.54	4.62	4.68	4.66	4.56
	SD	0.59	0.58	0.66	0.61	0.42	0.51	0.56	0.61	0.49	0.47	0.47	0.56
	P ( *vs* 84%)		*p* = 0.6701	*p* = 0.9365	*p* = 0.1598		*p* = 0.2021	*p* = 0.1605	***p* = 0.0051**		*p* = 0.5335	*p* = 0.6779	*p* = 0.5699
Joint Spaces	Average	4.35	4.34	4.36	4.2	4.88	4.8	4.81	4.71	4.9	4.79	4.88	4.72
	SD	0.57	0.62	0.65	0.58	0.36	0.43	0.42	0.48	0.35	0.43	0.3	0.45
	P ( *vs* 84%)		*p* = 0.9333	*p* = 0.9350	*p* = 0.1952		*p* = 0.3156	*p* = 0.3731	***p* = 0.0479**		*p* = 0.1638	*p* = 0.7597	***p* = 0.0278**
Contrast	Average	4.13	4.06	4.12	4.05	4.54	4.45	4.39	4.21	4.7	4.62	4.59	4.37
	SD	0.59	0.68	0.66	0.59	0.58	0.67	0.65	0.75	0.59	0.61	0.59	0.66
	P ( *vs* 84%)		*p* = 0.5837	*p* = 0.9365	*p* = 0.4994		*p* = 0.4744	*p* = 0.2263	***p* = 0.0156**		*p* = 0.5066	*p* = 0.3535	***p* = 0.0098**
Soft Tissue	Average	3.93	3.84	3.96	3.81	4.62	4.49	4.37	4.25	4.58	4.57	4.61	4.47
	SD	0.63	0.75	0.74	0.65	0.58	0.67	0.64	0.77	0.5	0.54	0.51	0.54
	P ( *vs* 84%)		*p* = 0.5174	*p* = 0.8277	*p* = 0.3509		*p* = 0.3021	***p* = 0.0434**	***p* = 0.0079**		*p* = 0.9237	*p* = 0.7671	*p* = 0.2932

### Inter-reader agreement

Mean interreader reliability in pelvis radiographs was 84.5%. Interreader agreement was slightly lower with at the 72.5% dose level in shoulder and at the 84% dose level in spine images. Interreader agreement over all 600 X-rays was 80%. The corresponding mean intraclass correlation coefficients for all images were substantial, with 0.796 (range 0.765–0.824).

## Discussion

Following the ALARA-principle, it is a constant challenge in radiology to maintain image quality with increasing dose reduction.^24^ In general, the applied radiation is significantly greater in computed tomography than in plain radiography.^25^ However, X-rays remain by far the most frequently ordered radiological examination. In 2014 alone, about 140 million X-rays were taken in Germany, of which skeletal images accounted for the second-largest share.^1^ In addition to technical advances in hardware, the introduction of digital radiography and image postprocessing opened up further opportunities for dose reduction. To ensure the preservation of diagnostic quality, the imaging effects of dose reduction must be subjected to quality controls.

Previous studies have already achieved remarkable reductions of ionizing radiation in conventional imaging. Kloth et al reduced dosage up to 42% in pelvis and to 37% in knee radiographs. Also, Jeon et al were able to decrease standard mAs to 50% in full spine X-rays. However, these studies were based on the visualization of narrowly defined parameters, such as bone-implant interface, implant-surface character or measurability of anatomic angles. These requirements were easily met even after significant dose reductions. Nevertheless, structural information and the diagnostic quality of these images beyond the narrowly defined parameters was oftentimes lost..^10–12,26,27^

The main finding of our study is the preservation of diagnostic quality and the readers’ respective diagnostic confidence in almost all radiographs. Ninety-eight percent (598 out of 600) postprocessed radiographs maintained clinical diagnostic reliability beyond gross anatomy, even after large dose reductions. As reported in previous studies, the readers were able to detect quality differences with respect to the applied dose.^28^ The statistical analysis confirmed this trend of superior overall performance at higher dose levels in all anatomic regions and dose groups. Significant differences in overall image quality were observed only in the lowest dose group in shoulder (*p* = 0.0055) and lumbar spine (*p* = 0.0192) radiographs. Nevertheless, all images in these two groups were rated as diagnostically sufficient to answer the clinical questions. In terms of the ALARA principle, precisely these images – obtained with the lowest dose and sufficient diagnostic quality – are superior to all others.

Furthermore, our results substantiate previously described shortcomings of soft tissue depiction in low dose skeletal radiography. In comparison to cortex, trabeculae and joint spaces, overall contrast and soft tissue visualization were the weakest image items throughout all anatomic regions and dose groups. Also, the only two images rated as minor in overall diagnostic performance revealed their main deficits in the depiction of soft tissue. This imaging parameter could become a limiting factor with further dose reduction. Therefore, it appears to be worthwhile to pursue existing approaches to enhance soft tissue depiction in skeletal radiography.^29,30^

In Germany, the dose reference values are defined as the 75th percentile of a distribution of patient doses from different users, including analog and digital systems.^19^ In our study median radiation for pelvis, shoulder and lumbar spine were minimized to 5.37 dGy*cm2, 0.46 dGy*cm2, 6.54 dGy*cm2, corresponding to 21.5%, 18.4 and 18.7% of the rDAP, respectively. The implementation of image postprocessing thus allows limiting the required dose to about one-fifth of the reference value. Given the broad clinical applicability of this technique, the existing reference doses should be carefully reconsidered, even although a further reduction in rDAP seems foreseeable with the eventual phasing out of analogue systems.

In radiography, the balance between image quality and applied radiation dose depends, to a certain extent, on the patient’s body shape. Normally, less radiation is needed in slim individuals to achieve sufficient quality.^31^ This is one of the main reasons why the rDAP is not a threshold value. The crucial point is that the mean values of the applied doses of an examination type at an X-ray unit should not exceed the corresponding reference value.^19^ Our study was designed to demonstrate broad applicability of the methodology in a typical clinical setting with a mixture of trauma an orthopedic in- and outpatients. Therefore, we enrolled patients consecutively without pre-selection criteria in terms of body composition, rather than sampling from a predefined population. This approach results in two study limitations: First, our study population is mainly representative of institutions like our own, even although the study demographics (age and gender).^2^ Second, we tolerated a certain level of imprecision regarding the relative dose reduction with deviations between the target and the actual dose levels. For instance, the median dose reduction between the 60 and 50% dose groups of pelvis radiographs was only 3.7% (reduction from 59.6 to 55.9%). These variations can be attributed to the fact that we examined 50 different patients in each of the dose groups. Examining the same 50 patients in four dose levels might have reduced these variances, but would have meant a significant reduction in patient numbers and thus a limitation of applicability and representativeness. Furthermore, the deviations mentioned above (*e.g.,* 3.7% *vs* 10%) only reflect the ratio of two group medians to each other and should not be misunderstood as a lack of actual dose reduction. Sufficient dose reduction for each individual is ensured by the AEC, which determines the required exposure time for each patient individually, depending on the body dimension, and switches off the X-ray generator when a preset relative dose value (84%, 71%, etc.) is reached. Additionally, our results are limited to the three examined anatomic areas. An application in other skeletal regions, such as the cervical and thoracic spine or the extremities (humerus, femur, etc.) appears promising, but remains to be investigated, especially with regard to soft tissue visualization.

In conclusion, postprocessing in skeletal radiography allows for a large dose reduction while maintaining clinically significant image detail. In fact, radiation doses were reduced to a minimum of 21.5%, 18.4 and 18.7% of the reference dose in pelvic, shoulder and lumbar spine radiographs, respectively. Even after this extensive dose limitation, diagnostic confidence was preserved across all dose groups. Therefore, our results suggest a broad application of postprocessed radiographs to ensure further dose reduction in clinical practice.
